# 

**Published:** 1894-11-17

**Authors:** 


**Notices of Births, Deaths, and Marriages** are inserted in this column at a charge of 2s. 6d. for an announcement not exceeding four lines.

**Notice to Advertisers and Subscribers.**

All Advertisements, Orders for Copies of The Hospital, and Business Communications should be addressed to **The Publisher, NOT TO THE EDITOR,** The Hospital, 428, Strand, W.C.

The Cost of Subscription, post free, is as follows:—Per week, 2½d.; per quarter, 2s. 8d.; per half-year, 5s. 3d.; per annum, 10s. 6d. Foreign:—Per Annum, 12s. 8d.; payable, in all cases, in advance.

### NOTICE TO CORRESPONDENTS.

All MS., Letters, Books for Review, and other matters intended for the Editor should be addressed The Editor, The Lodge, Porchester Square, London, W.

The Editor cannot undertake to return rejected MS., even when accompanied by stamped directed envelope.

**"Burdett's Hospital Annual," 1894.**

Fifth year of publication. 900 pp. Boards, gilt lettering. A most complete guide to British, Colonial, Indian, and American Hospitals, Dispensaries, Nursing and Convalescent Institutions, and Asylums. Price 5s. Published by The Scientific Press (Limited), 428, Strand, W.C.

**NOTICE.—The Index for Vol. XVI. (ending September 24th, 1894) is on sale at the Office of this Journal, Price 2½d., post free.**

### Scraps and Gleanings.

It is proposed to erect a central cottage hospital at Tondu, in Wales.

The Durham Board of Guardians are about to erect an infirmary for their workhouse.

The proposed site for the consumption hospital for Belfast adjoins the Annadale Manor Estate.

Mr. J. E. Wilson has succeeded Sir John Jaffray as treasurer to the Birmingham General Hospital.

The annual festival service of the Oxford Radcliffe Infirmary was held in the chapel on St. Luke's Day.

The foundation stone of an Infectious Diseases Hospital for Leith was laid recently. The cost is estimated at £35,000.

An isolation hospital appears to be much wanted at Newton Abbot. A local doctor reports twenty-two cases of typhoid since May last.

Mr. John Dawson, of Northbrook, has given £2,000 to the West of England Eye Infirmary. New buildings are under consideration.

It seems likely that the scheme of extending the Edinburgh City Hospital will be abandoned in favour of a new building on a new site.

The Bishop of Aberdeen and the Bishop of Southwark have become Vice-Presidents of the Church Society for the Promotion of Kindness to Animals.

It is stated that it was the doctor attached to the Imperial yacht the *Polar Star* who first discovered that the Emperor of Russia was suffering from an affection of kidneys.

Mr. Harben has promised to give £4,000 for the purpose of building a Convalescent Home near the seaside for the working men of Hampstead and Kentish Town.

Patients have largely increased at the Devon and Cornwall Ear and Throat Hospital, but the finances require strengthening, as an adverse balance is shown on the accounts.

The Barry district in Wales is to have a fever hospital, erected at an estimated cost of £6,000. A site has also been scheduled by the Local Government Board for a cholera hospital for the port of Barry.

The Lord Chief Justice has consented to preside at the festival dinner in aid of the funds of the City of London Hospital for Diseases of the Chest, to take place at the Hotel Metropole on April 30th next.

The meeting of the Royal Surrey County Hospital is already bearing fruit. Baron Henry de Worms has given a hundred guineas, and Lord Rosebery, Viscount Middleton, and the Bishop of Winchester have each given £25.

The Stranraer Cottage Hospital has now completed two years of a most prosperous existence. It is fortunate in creating so much interest in its neighbourhood as to secure all the necessary funds for its maintenance without difficulty.

It appears not improbable that the Chesterfield friendly societies may be instrumental in securing a new ward for the Chesterfield and North Derby Hospital. All honour to their efforts to care for their sick brethren if the scheme is carried out.

On the 18th ult. a new deaf and dumb institution was opened by the Duchess of Rutland. The founder of the original institution was Mr. Roe, who has devoted himself with great success to the work. The new home has cost £12,000, and at present contains about 70 inmates.

The master of a workhouse is not a universally popular person; it is all the more pleasant to record that at the Birmingham Workhouse Infirmary the master has been presented with a token of goodwill by the workmen, male attendants, and Managing Committee of the Infirmary.

In addition to his gift of £3,000 to the Devon and Exeter Hospital, and of £2,000 to the Eye Infirmary, Mr. John Dawson, of Northbrook, Exeter, has expressed his intention of contributing £3,000 to the Exeter Blind Institution, and £2,000 to the Dispensary, making altogether £10,000.

The Worcestershire County Council are wise in their efforts to promote amalgamation and centralisation when establishing fever hospitals within their jurisdiction. The fever hospitals about to be erected at Malvern and Bromsgrove are to combine with other districts, and thus a better class of hospital will be secured.

The Huntly Cottage Hospital adds another example, to the many we have instanced at different times, of the interest shown in the cottage hospital movement. The financial support is quite adequate for its needs. The Huntly Cottage Hospital had the difficult task of dealing with a certain number of infectious cases during the year.

Plans for a new wing at the Devon and Exeter Hospital are now under discussion, They provide for extensive alterations in the internal working of the hospital and arrangements of the wards, also a new wing on the central block of the patients' recreation ground. The alterations and additions will provide for a large number of beds.

At the last quarterly court of the Radcliffe Infirmary at Oxford the chairman stated that it was necessary to sell out £1,000 of the invested property to meet current expenses. A general feeling was expressed that the support received both from town and University was unsatisfactory. A large decrease on household expenditure was shown during the past quarter.

It is generally admitted that East Grinstead needs an isolation hospital. The scheme of establishing one appears likely to be carried out without the usual friction, opposition, and difficulties which attends such a course in most neighbourhoods. A likely site has been offered, and the Local Government Board are considering a proposal to borrow £3,500 to defray the cost of building.

The 112th anniversary of the Nottingham General Hospital was made the occasion to especially thank Colonel Seeley for his munificent gift of the house known as "The Cedars" as a convalescent establishment in connection with the general hospital. Besides other instances of Colonel Seeley's generosity, he has increased his subscription to the Convalescent Fund to £200 per annum.

A severe epidemic of typhoid and diphtheria is visiting Newport in the Isle of Wight. The Town Council have acted with some energy, and various means to stay the spread of the disease through defective drainage are being resorted to. The Hermite system is being adopted. It appears that Mr. Damant, inspector to the Local Board, foresaw, and has pointed out, the dangers existing in Newport but with no result.

### BOOKS RECEIVED.

HENRY RICHARDSON.
"Record of the Miller Hospital and Royal Kent Dispensary." By John Poland, F.R.C.S.

F. J. REBMAN.
"Personal Hygiene." By Mrs. Ada S. Ballin.

JAMES MACLEHOSE AND SONS.

"Characteristics and Embodiments of Life, with Special Reference to Pathology." By Joseph Coates, M.D.

MACMILLAN AND CO.
"Textbook of Anatomy and Physiology for Nurses." By Diana Clifford Kimber.
"Physiology for Beginners." By M. Foster, M.A., M.D., etc.

THE UNIVERSITY PRESS, CAMBRIDGE.
"A History of Epidemics in Britain." Vol. II. By Charles Creighton, M.A., M.D.

SMITH, ELDER, AND CO.
"The Vagabonds." By Margeret L. Woods.

METHUEN AND CO.
"Round the Red Lamp." By A. Conon Doyle.

**Periodicals and Pamphlets.**—Westminster Review, Provincial Medical Journal, The Corpuscle, Public Health, Verulam Review, Leeds Hospital Magazine, The Tri-State Medical Journal, Homœopathic Review, Proceedings of the Society for the Study of Inebriety, Children's League of Pity Paper, The Sun, The Planter, Summer Tours and Cruises, 1894, The Humanitarian, Child's Companion, Friendly Greetings, Light in the Home, The Cottager and Artizan, Our Little Dots, Cassell's Family Magazine, Cornhill Magazine, Guy's Hospital Gazette, English Illustrated Magazine, Girls' Own Paper, Boys' Own Paper, The Leisure Hour, The Sunday at Home, and Public Opinion.

### THE STUDENT OF MEDICINE.

### APPOINTMENTS.

R. W. D. M. Cameron, M.D., D.Sc.Edin., appointed Medical Officer of Health for the combined Counties of Wigtown and Kirkcudbright. H. E. Firth, appointed House Physician to the Leeds General Infirmary. J. W. Hainsworth, M.B.Vict., appointed Resident Obstetric Officer to the Leeds General Infirmary. J. A. Hicks, jun., M.R.C.S.Eng., L.R.C.P.Lond., L.S.A., M.P.C., appointed Honorary Physician to the Nazareth House Convalescent Hospital, Bexhill-on-Sea, Sussex. J. R. Kennedy, M.B., C.M.Aberd., appointed Medical Officer, Carradale, Kintyre, Argyleshire. T. D. Luke, M.B., B.Ch.Irel., appointed Junior House Physician to Smedley's Hydropathic Establishment, Matlock. W. McClelland, M.B., Ch.B.Vict., appointed Non-resident Medical Officer to the Gynæcological Department of the Liverpool Royal Infirmary. A. L. Mathieson, M.B., C.M.Glasg., appointed Medical Officer for the Grimesthorpe District of the Sheffield Union. W. Morris, L.R.C.P.Edin., L.F.P.S.Glasg., appointed Medical Officer for the Western Bolton District of the Bolton Union. L. Newton, M.R.C.S.Eng., L.S.A., reappointed Medical Officer for the Sawtry District of the Huntingdon Union. J. H. Pim, L.R.C.P., L.R.C.S Irel., L.M., appointed Medical Officer for the Blankney District of the Sleaford Union. H. E. Rayner, F.R.C.S Eng., appointed Surgical Registrar and Anæsthetist to the Hospital for Sick Children, Great Ormond Street. J. R. Richmond, appointed Medical Officer for the Overton and Penley District of the Ellesmere Union. A. S. Robinson, M.A.Cantab., L.S.A.Lond, appointed House Surgeon to the Leeds General Infirmary. G. A G. Simpson, M.R.C.S.Eng., L.S.A., appointed Medical Officer of Health to the Acton Local Board. P. C. Smith, M.A., M.D., D.P.H.Camb., appointed Medical Officer of Health to the Parish of Wandsworth. Mr. H. Steven, appointed Medical Officer for the Second District of the Warminster Union. W. Stoney, M.B.Vict., appointed Resident Medical Officer to the Ida Hospital of the Leeds General Infirmary. D. B. Trumper, M.B.Vict., appointed House Surgeon to the Leeds General Infirmary. F. Walker, M.R.C.S., L.R.C.P., appointed House Physician to the Leeds General Infirmary. W. Washbourn, M.R.C.S., L.R.C.P., appointed Assistant Surgeon to the General Infirmary at Gloucester and the Gloucestershire Eye Institution. A. Westlake, M.B., C.M.Edin., appointed Medical Officer of the Workhouse of the Grimsby Union.

### VACANCIES.

**Resident Medical Officers.**

East Sussex County Asylum, Hayward's Heath.—Junior Assistant Medical Officer. Salary £100 per annum, with board and lodging. Applications to the Medical Superintendent by November 20th.

General Hospital, Birmingham.—Assistant House Surgeon; must possess surgical qualification. Appointment for six months, but may be held by re-election for a further period of six months. Board, residence, and washing provided. No salary. Applications to the House Governor by December 1st.

Kent and Canterbury Hospital.—House Surgeon; must be a registered medical practitioner, unmarried. Salary £90 the first year, with board, &c., rising to £100 the second year. Applications and testimonials to A. J. Lancaster, Secretary, before November 23rd.

**THE STUDENT OF MEDICINE**—*continued.*

Hospital for Sick Children, Great Ormond Street, Bloomsbury, W.C.—Resident Medical Officer as House Physician. Appointment for six months. Salary £20 per annum, with board and residence; must be unmarried. Applications and testimonials to Adrian Hope, Secretary, before November 21st.

Hospital for Sick Children, Great Ormond Street, Bloomsbury, W.C.—Resident Medical Officer as House Surgeon. Appointment for six months. Salary £20 per annum, with board and residence; must be unmarried. Applications and testimonials to Adrian Hope, Secretary, before November 21st.

Liverpool Northern Hospital.—Assistant House Surgeon; doubly qualified. Salary £70 per annum, with residence and maintenance in the house. Applications to the Chairman by November 2[illegible]th.

**Non-Resident Medical Officers.**

Belfast Royal Hospital.—Assistant Staff-Surgeon and a House Surgeon for the Extern Department; doubly qualified. Applications to the Chairman of the Board of Management by November 17th.

Hospital for Women, Soho Square, W.—Registrar. Appointment for twelve months. Honorarium, 25 guineas. Applications to David Cannon, Secretary, by November 20th.

